# Nurses’ experiences of expanded responsibilities, independent decision-making and action in specialized outpatient palliative care: a qualitative interview study

**DOI:** 10.1186/s12904-026-02399-0

**Published:** 2026-10-30

**Authors:** Eva-Maria Regelmann, Sonja Owusu Boakye, Julien Pöhner, Henrikje Stanze

**Affiliations:** Faculty of Social Sciences, University of Applied Sciences Bremen, Bremen, 28195 Germany

**Keywords:** Expanded nursing roles, Autonomy in nursing practice, Palliative care

## Abstract

**Background:**

Given an increasing demand for patient care, healthcare systems worldwide focus on expanding nursing practice. Nurses in specialized outpatient palliative care (SOPC) take on expanded responsibilities requiring independent clinical decision-making and action, while little is known about how nurses themselves experience this practice. Therefore, this study aims to explore how nurses experience these aspects of their practice when delivering care in complex healthcare situations.

**Methods:**

A qualitative interview study was conducted with nurses who had current or previous work experience in SOPC (N=20) between June and August 2025. Data were collected through individual interviews, following a semi-structured format. Data analysis was guided by grounded theory methodology according to Strauss and Corbin. Strauss’s coding paradigm was used to develop an explanatory model of the phenomenon and its underlying conditions.

**Results:**

The core phenomenon identified is “responsibility borne in relationship-based autonomy.” Nurses describe being able to make decisions and act independently while remaining embedded in trusting interprofessional relationships and supportive team structures. The core phenomenon is influenced by the need to respond promptly to patients’ healthcare needs while applying and developing nursing competences. Intervening conditions include attitudes toward autonomy in nursing practice, self-confidence, and trust from colleagues, physicians, patients, and families. Nurses respond through strategies such as engaging in interprofessional collaboration to promote multi-perspective dialogue, applying specialized knowledge and work experience, and establishing direct contact with patients to assess and manage care situations. Consequences include experiencing meaningful work, perceiving improvements in care quality, and navigating ambivalence between professional empowerment and stressors. Contextual factors include managing complex care situations in patients’ homes, understanding patients’ situations holistically, and being strongly committed to patients and their families.

**Conclusion:**

Nurses’ experiences of expanded responsibilities performing independent decision-making and action in SOPC suggest that professional autonomy emerges within a relational and collaborative context. Confidence in one’s professional competence, interprofessional recognition, trust, and supportive organizational structures appear important for feeling safe when exercising professional judgment. Consequently, efforts to create the relational and organizational conditions that enable nurses to exercise their expertise safely and effectively are recommended.

**Supplementary Information:**

The online version contains supplementary material available at https://doi.org/10.1186/s12904-026-02399-0.

## Background

Nurses play a pivotal role in palliative care and constitute the largest professional group involved in the care of patients with life-limiting illnesses and their families, addressing the needs of an estimated 56.8 million people worldwide annually [1–3]. Their scope of practice extends beyond physical care to the assessment, management, and coordination of complex physical, psychosocial, emotional, and spiritual needs [3, 4]. Through their continuous and frequent contact with patients, nurses are well positioned to build trusting relationships, identify unmet needs, advocate for patient preferences, and facilitate communication within interdisciplinary teams [4, 5].

A central aim of palliative care is the alleviation of suffering and the promotion of quality of life throughout the illness trajectory [6]. Accordingly, nurses support patients and families in coping with uncertainty, symptom burden, progressive functional decline, and end-of-life decision-making [7], while serving as a bridge between patients, relatives, physicians, and other healthcare professionals to ensure continuity of care and support shared decision-making [7, 8]. The growing complexity of palliative care requires advanced clinical competences, including ethical consultation, patient-centred communication, and care coordination [8–10]. Education and professional development are therefore essential prerequisites for high-quality palliative nursing practice. Research has shown that nurses with specialized palliative care training contribute to symptom management, care coordination, patient safety, family support, and person-centred care [11, 12].

Given their role in palliative care, nurses are increasingly required to make complex clinical judgments and respond to rapidly changing patient needs. This involves taking on expanded responsibilities [5] and making decisions and acting independently within nursing practice [12–14]. While autonomy refers to nurses’ authority and responsibility to make decisions based on professional expertise [13, 14], independence describes the practical ability to act without the immediate presence or supervision of other healthcare professionals, such as physicians [14]. International evidence suggests that nurses taking on expanded responsibilities, having greater decision-making authority and a more proactive role in patient care can improve access to care [15] and enhance patient satisfaction [16]. Evidence suggests that expanded nursing roles in hospital and outpatient settings may also promote more efficient use of healthcare resources and reduce physicians’ workload, particularly in contexts characterized by high complexity and workforce shortages [16, 17].

In Germany, taking on expanded responsibilities and performing independent decision-making and action are integral components of nursing practice in specialized outpatient palliative care (SOPC). The German guideline on the prescription of SOPC [18] states that nurses require the authority to adapt interventions to patients’ individual needs, thereby enabling them to address rapidly changing care needs. Consequently, nurses are actively involved in symptom management, care coordination, and patient and family counselling [19–22]. Managing these highly complex situations requires specialized palliative care education and professional development [22–25], while care is delivered in patients’ homes within a 24-hour on-call system [20, 25]. In SOPC in Germany, the prescription of medication remains the responsibility of physicians [22], while nurses play an important role in continuously assessing patients’ symptoms and identifying when prescribed treatment may need to be adjusted. Thus, their clinical assessment provides an important basis for subsequent treatment decisions. Medication-related nursing activities are performed within this physician-defined treatment framework and are distinguished from independent prescribing or modification of medication prescriptions.

Research indicates that expanded nursing roles, such as within advanced nursing practice, are associated with challenges, including the need to balance increased professional independence with the responsibility accompanying such roles [26–28]. Specialized education, professional experience, interprofessional collaboration, organizational support, and trust in nursing competence have been identified as important prerequisites for taking on expanded responsibilities and performing independent decision-making and action [28, 29]. However, little is known about how nurses themselves experience the ability to make independent decisions and take action in practice and what is required to support this ability. SOPC provides a particularly relevant setting for exploring these experiences, as it enables nurses to expand both their knowledge and scope of practice.

Therefore, this study aims to investigate how nurses working in SOPC in Germany experience taking on expanded responsibilities and engaging in independent decision-making and action. Accordingly, the following research question was formulated:

*How do nurses working in German SOPC experience taking on expanded responsibilities*,* including independent decision-making and action?*

## Methods

### Design

Given the explanatory nature of the research question, a qualitative study design was adopted [30]. To gain an in-depth understanding of nurses’ experiences of taking on expanded responsibilities, including independent decision-making and action, and to develop an empirically grounded framework, the data analysis was guided by the principles of grounded theory according to Strauss [31] and Strauss & Corbin [32]. These principles included constant comparison and the iterative development as well as refinement of concepts and categories during the analytical process [32]. Through this process, an explanatory model of the phenomenon under study was developed. The study is reported in accordance with the COREQ reporting guidelines [33].

### Participants and recruitment

Eligible participants were nurses aged 18 years or older with sufficient German language skills who had completed vocational nursing training and had current or previous work experience in SOPC. No minimum duration of nursing or SOPC work experience was required.

Study information was sent throughout Germany via e-mail distribution lists of the German Society for Palliative Medicine (Deutsche Gesellschaft für Palliativmedizin) in May 2025. Simultaneously, four palliative care and two hospice services in the federal states of Bremen and Lower Saxony (due to the researchers’ geographical location) were contacted via telephone by the first author (EMR). Study information was provided to the service coordinators requesting to share it with nurses employed.

Nurses could contact EMR directly by e-mail or telephone to obtain detailed information on participation. No financial or non-financial incentives were provided for participation. After a two-week recruitment period, 24 nurses from all over Germany expressed interest in participating and met the inclusion criteria. Written informed consent was obtained after participants received information about the study, data processing, privacy, and voluntary participation.

The study received ethics approval from the Ethics Committee of the German Society of Nursing Science (application no. 24 − 007). All study documents, including information on informed consent, data collection, and data processing, were reviewed and approved as part of the ethics review process. The study was planned and conducted in accordance with the requirements of the European Union General Data Protection Regulation (EU GDPR) and under the guidance of the data protection officers of the conducting research institution and the participating university hospital.

Of 24 interested nurses, two did not respond further and two were unable to attend their scheduled interviews. Thus, a total of 20 nurses (Table 1) participated in an individual interview.

**Table 1:** Characteristics of the participants (*N* = 20)

Characteristic	Number of interviewees (*N*)
Gender	
Female	14
Male	6
Divers	-
Age (years)	
25–35	1
36–45	3
46–55	10
56–65	5
> 65	1
General school-leaving qualification	
Intermediate secondary school-leaving qualification	6
Entrance qualification for universities of applied sciences	5
General higher education entrance qualification	9
Professional experience in nursing (years)	
11–20	10
> 20	10
Professional experience in SOPC (years)	
0–5	4
6–10	5
11–20	9
> 20	2
Professional roles in SOPC^*^	
Leadership responsibilities	6
Education and training	7
Practice supervision	5
Case management	1
Wound management	1
Competences in palliative care^*^	
Certified palliative care nurse	20
Master’s degree in palliative care	1
Studying for a master’s degree in palliative care	2

*SOPC *specialized outpatient palliative care

^*^Categories are non-mutually exclusive

The sample is characterized by a high level of professional experience and all interviewees are certified palliative care nurses, as required by the German regulatory framework for SOPC. In addition, interviewees have professional experience across a wide range of clinical fields, including intensive and emergency care, geriatrics, internal medicine, and oncology. The high level of specialized knowledge is also reflected in several additional professional roles held within SOPC, e.g., leadership and case management.

### Data collection

Data were collected through individual interviews between June and August 2025. A semi-structured interview guide incorporating predefined topics and narrative prompts (see Additional File 1) was developed by the research team (EMR, SOB, HS). Narrative prompts and open-ended questions were used to encourage participants to recall and describe their experiences in detail [34]. The interview guide addressed predefined topic areas related to the research question, including interprofessional collaboration, expanded nursing responsibilities, independent decision-making and action, delegation, and responsibility. These topics served as a priori domains to guide data collection, while the analytical categories and their relationships were explored during data analysis. No modifications were made to the interview guide during data collection in response to emerging categories. The interview guide was pilot-tested by EMR with a nurse working in SOPC, whose data were not included in the final dataset. As the pilot did not indicate a need for modification, no changes were made to the interview guide.

Participants were recruited using convenience sampling [35], as eligible nurses who responded to the study invitation and volunteered to participate were included based on predefined inclusion criteria.

Data saturation was assessed by EMR, drawing on the concept of meaning saturation [36]. The assessment considered the emergence of new categories, additional dimensions or nuances within existing categories, and changes in the relationships between categories. From interview 15 onwards, no substantially new insights emerged, while the remaining five interviews further elaborated existing categories. After 20 interviews, EMR therefore determined that the data were sufficiently rich to address the research question. This assessment was documented by EMR and discussed within the research team, and the decision to conclude data collection was reached by consensus. All interviews were conducted and recorded by EMR, who had no prior relationship with any of the participants. The mode of recording depended on the interview format. Accordingly, interviews conducted via Zoom were recorded with video and audio, whereas only the audio track was used for transcription.

Participants could choose between in-person and online participation via Zoom or Microsoft Teams. Access to online meetings was restricted to EMR and participants, and recording began only after verbal consent had been obtained. Recordings were used solely for transcription and securely stored on external devices inaccessible to unauthorized persons. The interviews lasted 41–82 min (mean: 65 min). One interview was conducted in person at a hospice in northern Germany, while 19 were conducted via Zoom.

### Data analysis

Interview recordings were transcribed using an automatic speech-to-text tool^1^ performed locally on a password-protected device. EMR reviewed the transcripts verbatim and added the transcription rules according to Rosenthal [37]. The analysis process was supported by memo-writing and field notes capturing theoretical considerations [34].

The coding process consisted of three stages: (1) open coding, (2) axial coding, and (3) selective coding. During open coding, the data were broken down into discrete segments and assigned initial codes to identify relevant aspects of the phenomenon [32]. During axial coding, codes were grouped into categories and relationships between these categories were explored using Strauss‘s coding paradigm, considering causal conditions, context, intervening conditions, strategies, and consequences [31].

Subsequently, selective coding was used to integrate and refine the categories and their relationships to further elaborate the emerging explanatory model of the phenomenon. During the coding process, data segments were continuously compared with one another, as well as with existing codes and emerging categories. This process supported the refinement of codes and categories and the identification of similarities, differences, and relationships within the data [31, 32].

All transcripts were coded by EMR, who presented and discussed preliminary codes, categories, and their relationships within the research team throughout the analytical process. Divergent interpretations were discussed and resolved through consensus. The team included a sociologist (SOB), a nursing scientist with expertise in qualitative research (HS), and a nursing scientist with clinical expertise (JP).

The first author (EMR) holds a Master’s degree in Applied Therapeutic Sciences and has a professional background as a speech and language therapist. Prior to her enrollment in a PhD programme, she had worked in palliative care settings, where she collaborated interprofessionally with nurses and palliative care physicians. This professional experience provided familiarity with interprofessional palliative care practice.

Reflecting on the potential influence of the researcher’s professional background, EMR’s previous experience of interprofessional collaboration in palliative care settings may have shaped her attention to issues of professional roles, responsibilities, and interprofessional collaboration during the interviews and subsequent interpretation of the data. Further, the study was embedded within a research project focusing on nurse-led care in Germany, which may have directed attention toward expanded nursing responsibilities, independent decision-making, and opportunities for greater nursing involvement in patient care. This may have influenced the development of the interview guide as well as the interpretation of participants’ quotations.

EMR participated in a workshop^2^ on grounded theory to support the practical application of its methodological principles. Within the workshop, selected findings were discussed with external researchers while ensuring the protection of participants’ personal data. As the interview transcripts were originally in German, quotations were translated into English by EMR and subsequently reviewed and revised by a native English speaker with a PhD in Health Science. In this process, particular attention was paid to preserving the original wording and its intended meaning as closely as possible.

Data analysis and structuring were supported by MAXQDA (Version 24.11.1).

## Results

**Fig. 1:**
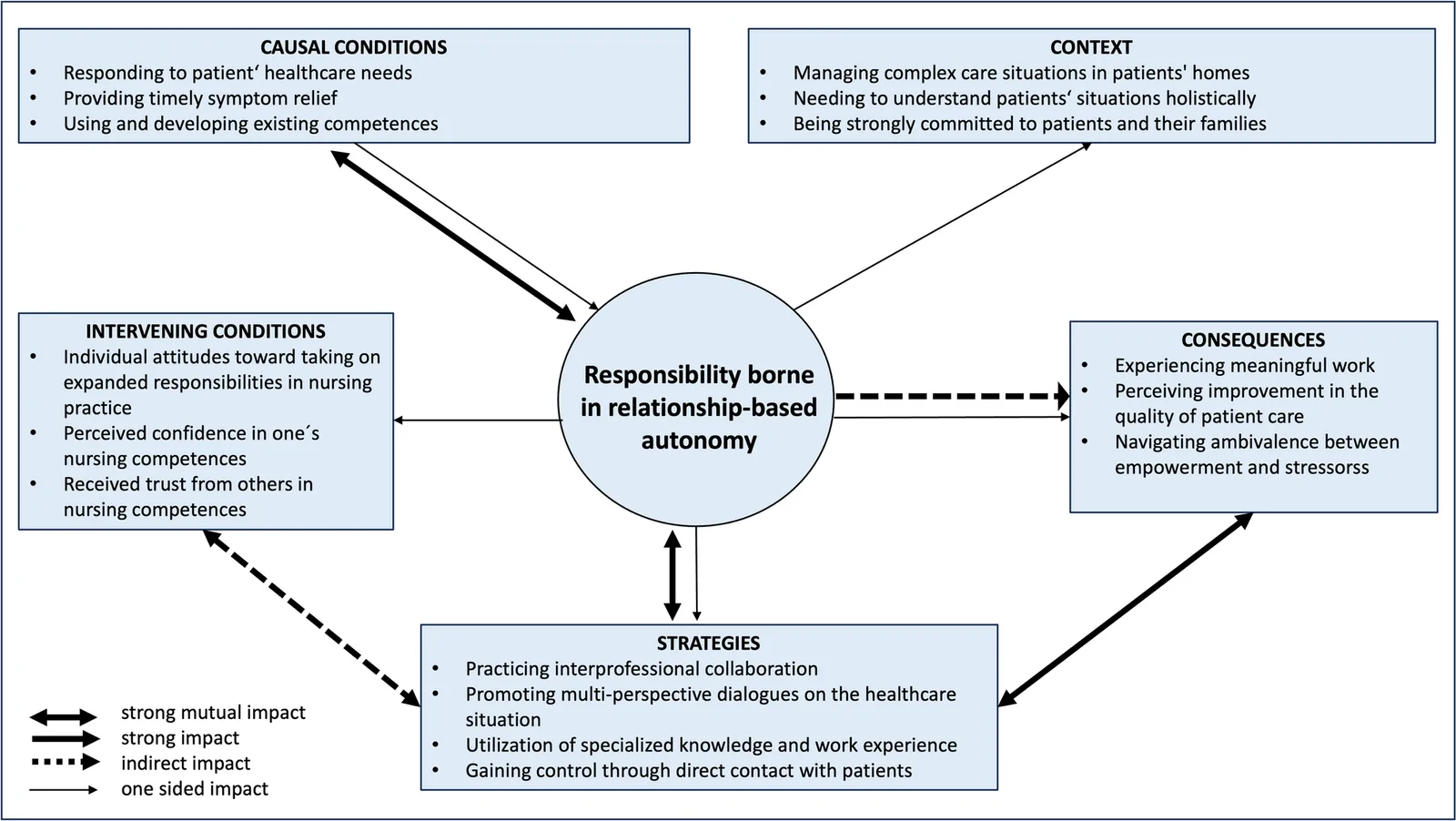
Experiences of autonomy among nurses working in SOPC

Using the coding paradigm according to Strauss [31], the core phenomenon and related categories were identified (Fig. 1). Accordingly, findings are structured around the core phenomenon, linking its causal conditions (what leads to a phenomenon), context (specific conditions in which it occurs), intervening conditions (broader influences), strategies (how people respond to the phenomenon), and consequences (the outcomes of these responses).

The core phenomenon associated with taking on expanded responsibilities and performing independent decision-making and action appears to be experienced as “responsibility borne in relationship-based autonomy.” Autonomy was not predefined as an analytical category but emerges through the integration of the central categories and their relationships within the coding paradigm. This integration reveals that the challenges associated with taking on expanded responsibilities and engaging in independent decision-making and action are experienced as manageable when nurses are able to seek reassurance within their team. Autonomy should therefore be understood as a concept in which nurses’ ability to take on expanded responsibilities based on their professional competences and to perform the associated tasks is supported by their embeddedness within the team. Delegation and consultation with physicians could occur within this relational context but were not, in themselves, interpreted as expressions of autonomy.

### Core phenomenon: responsibility borne in relationship-based autonomy

“Responsibility borne in relationship-based autonomy” emerges as the core phenomenon, integrating different dimensions of autonomy and independence described by nurses in SOPC. Across these dimensions, autonomy is experienced in relation to others and is closely linked to independent decision-making and action. Particularly important in this context appears to be the willingness to assume responsibility for these decisions and actions when physicians are not directly present to provide immediate supervision. Trust, professional confidence, and interprofessional collaboration shape how autonomy is experienced and enacted in SOPC. The core phenomenon appears to reflect an awareness of the impact of nurses’ decisions and actions on patients, particularly regarding symptom relief. Benefits, risks, and potential consequences are continuously considered throughout the care process. Interviewees emphasize that unconditional support and trust within the team create a safe environment for open communication. This strengthens their confidence in their own abilities and empowers them to make independent decisions:

And then, of course, there’s the support from the team-the absolute sense of security (.) I remember my very first day working in the SOPC team, and the senior physician said to me, ´No matter what decisions you make out there, I’ll stand behind you` (.) and right from the first day, he said, ´Whatever questions you have, just get in touch, and you should never do anything that doesn’t feel right to you (.) if something doesn’t feel right, let us know immediately`, that was such a relief for me and that’s something that has remained constant (.) the team really is a major cornerstone. (P5)

Support from the interprofessional team, particularly physicians who provide backup when needed, enables nurses to act independently in complex or critical situations. The opportunity to consult the physician and receive confirmation of their clinical judgment provides important reassurance for nurses in decision-making:

I had a crisis call while on the on-call service, where I was called out alone as a nurse to the patient or the relatives (.) and he was in the terminal phase, which was restless (.) that is, he was in the dying process (.) and despite the medication that we had already started, he was visibly restless and agitated […] I certainly already had some ideas from my own experience, however, for the consultation and also for my own reassurance, I then contacted the doctor and discussed the further course of action (.) and in the end this patient died during the night in my presence, well and with his symptoms under control […] and, um, also just for me to know, I have sound knowledge, or I can, it is available to me, I can access it (.) and then to also get the confirmation from the physician, ´Yes, that’s how we’ll do it, that’s a good idea`, or ´We’ll add that as well, that’s fine`. (P13)

Interviewees describe the interprofessional team as an important source of security. Accordingly, it seems that the confidence nurses need for independent decision-making and action arises from the sense of security created through trusting interprofessional collaboration.

### Causal conditions

Causal conditions are circumstances or factors that contribute to the emergence of the core phenomenon and describe the conditions under which it becomes salient. Within the coding paradigm, these relationships are understood as analytical relationships and do not represent causal effects investigated in this study.

#### Responding to patients’ healthcare needs

An important factor underlying the core phenomenon is nurses’ sense of responsibility for patients’ health and well-being. As they are often the first professionals to assess patients, typically before physicians arrive, the need for independent decision-making and action arises spontaneously according to the patient’s condition, despite certain tasks being delegated in advance by physicians:

So I would distinguish between specific procedures like port punctures and blood draws, that’s discussed during the assignment of tasks, and then those tasks are delegated (.) I think that makes the distinction easier (.) and otherwise, I think there’s an aspect that just happens (.) even when we set out for what’s supposed to be a routine visit, we’re often surprised to find that it’s anything but routine. (P12)

In situations of high symptom burden, nurses emphasize the necessity to act immediately to provide timely symptom relief. When medication is already included in an existing medication plan, nurses describe independently assessing the situation and deciding when its administration is required. So during acute episodes of pain and distress, immediate decisions are necessary to ensure timely symptom relief and avoid delays associated with attempting to contact a physician:

Um, last year I had a patient (.) it was an emergency, and he had severe shortness of breath, and first I gave him a tablet, Lorazepam, for this shortness of breath (.) that was listed in the as-needed medication plan […] but that didn’t help, then I gave him an injection, morphine, that was also listed in the medication plan, but it was still already the second medication that I had simply given on my own initiative […] especially the speed with which I administered something was a decision I made on my own initiative, but one that I then also confirmed in the phone call [with the physician], and that was completely okay, because anything else would simply have been torture for the patient. (P9)

It is suggested that independent decision-making and action are not goals in themselves, but rather result from nurses’ sense of responsibility to ensure timely, needs-based patient care. Consequently, decisions that go beyond the predefined treatment (e.g., regarding the intervals between individual doses as part of medication regimens) are not made arbitrarily, but are apparently based on clinical necessity in order to avoid delays in treating symptoms.

#### Using and developing existing competences

Another factor underlying the core phenomenon seems to be nurses’ desire to apply and further develop their competences. Seeking recognition of their professional expertise, nurses actively contribute to clinical decision-making and continuously expand their expertise through professional development, reflective practice, feedback, and ongoing learning:

The ability to assess situations, that is a major prerequisite for me, to bring that with me, and then, well-that I always have this willingness and enjoyment in continuing to develop myself (.) that I, so to speak, want to keep learning, that I am also happy to be open (.) when someone says, ´Oh, I would have done that differently`, or ´How did you come to that approach`, so that I am happy to let myself be corrected (.) that I bring that kind of openness with me (.) so that, in the interests of the patients, a good outcome is achieved. (P5)

Overall, independent nursing decision-making appears to be a dynamic, reflective process shaped by continuous learning and interprofessional exchange rather than fixed professional knowledge.

### Intervening conditions

Intervening conditions are factors that influence how individuals respond to a phenomenon through the use of strategies.

#### Individual attitudes toward taking on expanded responsibilities in nursing practice

Nurses’ attitudes toward taking on expanded responsibilities in nursing practice appear to shape how they experience its implementation. Interviewees describe both positive and critical aspects of independent decision-making and action and tasks delegated by physicians. A positive attitude is reflected in participants’ confidence and willingness to take on new tasks, including those traditionally associated with medical practice:

So the motto is […] not being able to do something is no reason not to do it, in the context of patient care (.) so it is always about what the problem is and why physicians think they need to ask me to do something, um, whatever it may be (.) I listen to that first and, of course, critically ask, ´Why should I do this now?​` (P2).

While tasks delegated by physicians are largely perceived as a positive expansion of nursing practice, concerns are also expressed regarding legal uncertainties associated with delegated tasks. In particular, interviewees describe ambiguities regarding which tasks may be delegated to whom and under what conditions:

Especially since we don’t really have a clear legal framework, and even when there is a legal framework, there are still grey areas where it is not entirely clear who is allowed to do what, how, and under which circumstances (.) it is simply unclear for many of these delegated tasks who is allowed to delegate what to whom, and why and how. (P8)

Taken together, these statements indicate that expanded nursing responsibilities are sometimes practiced in situations in which the boundaries of delegation and the formal authorization of specific nursing actions are perceived by nurses as unclear. Interviewees’ statements also reveal variation in how nurses respond to such uncertainty and in their willingness to take on responsibility for independent decisions and actions. While some nurses describe confidence in making and justifying independent decisions, it is also indicated that differences in the willingness to make such decisions create pressure among colleagues:

So the patient should feel comfortable and have the sense that, ​my problem is being taken seriously, and someone is making a decision that brings me relief […] but that also makes colleagues feel under pressure: ​If one person does something, then I have to do it too​. (P2)

Uncertainty toward independent decision-making and action among colleagues may also influence nurses’ own attitudes and perceptions:

Somehow, I’ve always got that in the back of my mind now, ever since two colleagues developed this incredible need for reassurance and said, ´No, I’m not lifting a finger until a physician has signed off on it` […] and that was something I wasn’t familiar with before, because I don’t do anything without thinking it through (.) and I can somehow justify everything I do (.) if I couldn’t, I wouldn’t do it […] but when I think about my colleagues in a situation like that [in independent decision-making and action], it sometimes makes it difficult for me (.) not because I’m actually afraid myself, but it holds me back. (P7)

Overall, taking on expanded responsibilities in nursing practice is largely regarded as an important professional achievement and innovation in nursing care:

So, you really have to say, over the last 17 years, what we have built up there, the issue of working on an equal footing, the issue of independently administering medication without having to consult physicians, um, we have simply built up a great deal (.) this issue of what diagnostics the nurse can also carry out, we are always in communication with one another. (P11)

Similarly, for most of the interviewees, performing tasks delegated by physicians independently and assuming expanded areas of responsibility are routine parts of daily practice:

So, I think, we are already very, we do many things that would officially be medical tasks, and where perhaps sometimes I am not even that aware of it anymore, because I have simply been in this, in this field for such a long time (.) nevertheless, I am aware that prescribing medication, for example, is a medical task (.) actually regardless of which medication it is. (P9)

The most commonly expressed positive view of expanded responsibilities is the potential to enable timely interventions and more efficient care in accordance with existing agreements with physicians. This is seen as particularly important in situations requiring rapid symptom control, where nurses perceive immediate action as contributing to symptom relief and patient stability:

So, I feel comfortable when I can carry out delegated tasks directly on site (.) that-, that is, for example, administering morphine in an emergency situation or in a situation of shortness of breath or pain (.) I think that is good and important and right, that I can, so to speak, take on that task in order to act quickly. (P3)

As tasks delegated by physicians become routine and no longer consciously perceived as role extensions, it seems that trust in nurses’ competence becomes embedded in everyday practice. Consequently, the normalization of expanded nursing roles also appears to reflect implicit interprofessional trust.

#### Perceived confidence in one’s nursing competences

The level of confidence that nurses have in their own competences helps them feel safe and comfortable when taking on expanded responsibilities. Consequently, perceived confidence appears as a crucial factor that provides nurses the opportunity to perform tasks delegated by physicians:

I am, for example, responsible for the dosage, for the infusion rate (.) per hour (.) so how many milligrams per hour of the medication the patient then receives, at the moment when I start the pump (.) because the physician is, after all, not standing next to me, and the phone call has ended (.) so he has the deepest trust that I will carry it out exactly as he prescribed […] I simply have a great, great deal of experience with that (.) and I have no problems with it at all. (P15)

From the nurses’ perspectives, confidence in their competence is based on specialized knowledge and supports independent decision-making and action as well as the willingness to take on responsibility:

I always say, knowledge is power (.) we simply have to train ourselves in these areas, and of course what gives me confidence is, firstly, my palliative care training […] but of course also the many years of experience that I have […] so it helps me tremendously, because I know what I have to do in which situation (.) when I am confident in what I am doing, then I can also take responsibility for it and do it with a clear conscience. (P11)

Consequently, palliative care expertise appears to strengthen professional self-confidence through awareness of one’s own competences. At the same time, recognizing these competences is considered important for avoiding practice beyond one’s professional limits:

I think I have very good clinical observation skills (.) I think I have very good professional expertise in my field (.) I think I can reflectively distinguish the boundaries of things that are not my field, so that I only take on responsibility where I have the qualification. (P10)

These statements also suggest that professional self-confidence not only enables nurses to take on more responsibility, but also helps them define the limits of their competences. Self-confidence is thus also described as the ability to accurately assess one’s own competences and limitations as a nurse, which suggests that it also functions as a form of professional self-regulation.

#### Received trust from others in nursing competences

Trust that others involved in the care process place in nursing competences appears essential for taking on expanded responsibilities. It seems to reflect confidence not only in nurses’ professional competence but also in their ability to recognize their own limits and seek support when needed:

When there was trust (.) when there was trust, not only in my practical skills, but also in my ability to judge how far I was allowed to go (.) that is, when I knew that they could rely on me to get in touch when things were critical, then I was able to act well (.) yes. (P20)

Trust within the interprofessional team emerged as an important condition for taking on expanded responsibilities, providing security, encouraging open communication about uncertainties, and legitimizing support-seeking in challenging situations:

The way we interact in my team, in our team (.) so really this relationship of trust, this feeling of being supported, I would put that right at the top (.) and that today I can give myself permission, I would say I gave myself that permission a few years too late, I do not have to have an answer for every situation (.) and I also do not have to respond in every situation that noticeably overwhelms me (.) I am allowed to say, ´I am overwhelmed, I want to do justice to you and your situation` (.) I would now, for one reason or another, like to bring someone else in, and even if that is met with a lack of understanding, I have that right. (P12)

Trust from patients and their families appears to develop over time through nurses’ approachability, willingness to help, and expertise. Ongoing contact throughout the care process further strengthens trust in their professional recommendations and competence:

As a nurse, you are, after all, very close to the patient, and my experience actually shows that the patient’s trust somehow, well, they simply do not question it at all, because for them it is clear, okay, she is competent, she has already helped me over the last few days here (.) if she is of the opinion that this has to be done this way now, then that is how we do it. (P18)

Further, it is suggested that supportive and trusting interactions appear to create a calmer working environment, which in turn reinforces professional self-confidence and facilitates independent action:

What supports me? So, I would say, when the patients or the relatives let me do it (2) trust what I am doing (.) then that is reflected back to me, and I feel more comfortable in the situation. (P9)

Trust from physicians appears particularly important, developing through long-term collaboration and demonstrated nursing expertise within established organizational structures. Recognition of nurses’ professional competence seems to reduce the need for constant justification and facilitate expanded responsibilities in autonomous practice. Further, trust placed in nurses by physicians is mentioned as a significant source of relief allowing nurses to openly communicate uncertainties without worrying about judgment:

The trust of the physicians (.) that they simply know that when I am there on site, I can really assess the care situation well (.) and that what I say is also reliable for them (.) that is trust and working on an equal footing, absolutely, and also being able to express my own uncertainty (2) so that I can also say, ´I’m just not really sure right now`, that is part of it as well. (P17)

In the absence of established professional relationships, nurses actively seek to build trust with physicians to support autonomous practice. This trust is described as developing through demonstrated competence, responsiveness to individual expectations, and shared reflection on professional decisions. Greater trust from physicians appears to strengthen nurses’ opportunities for independent decision-making and action, while recognition of nursing expertise provides professional validation:

A basic trust that is simply there (.) one of our female doctors resigned and today was her last day, and she then said that she had learned a lot from me (3) I was a little embarrassed about that, but things like that are just coming out now (.) I would never have thought before that something like that existed. […] this close relationship with the nursing staff, the doctors always emphasise that as well and experience it as something completely enriching. (P19)

Overall, perceived and received trust in nursing competence appears to strongly shape the core phenomenon by influencing the scope for independent decision-making and action granted to nurses and their willingness to assume the associated responsibility. The findings point to a broader understanding of professional competence, in which knowing when to involve others is considered as important as acting independently. A trusting team environment appears to foster psychological safety, enabling nurses to acknowledge uncertainty and seek support without perceiving this as professional failure. In this sense, recognizing one’s own limits and drawing on the expertise of others emerges as an integral part of competent professional practice.

### Strategies

Strategies relate to the behaviours, actions and approaches to interaction used by nurses working in SOPC to respond to and manage the phenomenon. These strategies can explain how nurses actively deal with challenges, responsibilities, and situations in their professional practice.

#### Practicing interprofessional collaboration

Nurses view interprofessional collaboration in SOPC as integral to their work, serving as the foundation of high-quality care and a means of managing complex situations holistically. Interprofessional dialogue appears to strengthen independent decision-making by integrating diverse expertise and perspectives to support the best possible and most sustainable care:

I’m just thinking quite spontaneously of a wound situation […] and for me, um, consultation was necessary, and I, um, with the relatives’ consent, was allowed to take a photo by the quickest route (.) I was able to send it directly to our, um, wound care manager, and, um, I really received a call back within ten minutes and the wound care manager’s assessment of whether, um, the approach I had chosen was okay or what she would do differently, what else she would point out (.) and for me, these are very important building blocks in our SOPC team, which lead to success very quickly, where I, the relatives, the patient above all, have safe, reliable, and the best possible care at home (.) and where, by the shortest route, I have a wide range of qualified people in my team, a multidisciplinary team, yes. (P5)

Interprofessional collaboration and promoting multi-perspective dialogues are valued, but also practiced through regular interprofessional team discussions. These meetings facilitate collaborative decision-making, care coordination, and emotional support in challenging care situations:

So, what is positive is that every four weeks we have a palliative care meeting, where physicians and nursing staff sit down together and take another look at particular cases, um, critical cases, and look at them together […] and sometimes we also discuss critical situations (.) a young mother with young children, for example, so something that is also emotionally burdensome, where this is then discussed with the doctors and we look at how we can stabilise the team, how we can deal with the situation, and, um, what more we can do for the family. (P2)

Overall, interprofessional collaboration appears valuable because it provides a more comprehensive understanding of the care situation. At the same time, collaborative reflection appears to strengthen confidence in clinical decision-making by validating nurses’ judgments and distributing responsibility across the team.

#### Utilization of specialized knowledge and work experience

Another strategy is the utilization of specialized knowledge and professional experience. These appear to provide nurses with a sense of security in complex palliative care situations and support effective responses to challenges. Professional competence is understood not as static knowledge, but as an ongoing process of acquiring, updating, and integrating new insights into practice:

Professional competence that I have simply acquired over the years now and also continue to acquire for myself (.) so I also regularly read studies and new findings, so that I think I am also at quite a good level, also through continuing education and further training. (P9)

Specialized knowledge appears to be applied primarily to accurately assess patients’ care needs through continuous evaluation of their condition and appropriate interventions. Nurses also describe interprofessionally developed assessments as helpful and repeatedly refer to the use of so-called whitelists in critical situations. Compared with procedural guidelines such as standard operating procedures, whitelists are perceived by interviewees as less complex and more flexible. Rather than being intended for standardized application across all patients with the same condition, whitelists are adapted to the individual patient and their specific care situation, allowing nurses to act without requiring immediate consultation with a physician. Nurses report that whitelists are developed within SOPC teams and authorized by physicians, providing an agreed framework for nursing actions in defined clinical situations. Participants describe these arrangements as enhancing their sense of security and providing a reliable basis for clinical decision-making:

So, a whitelist is, well, there are many different areas for it (.) so, in the end, it is like a flowchart, where you have a problem situation and a fixed portfolio of measures (.) that does not, um, necessarily have to be medication-related, although of course it is that as well (.) it can also be treatment measures that you then apply, so to speak, as a cascade, without having to, because these are exceptional situations or emergencies, go into extensive consultation [with physicians] (.) you can then act directly when the person in front of you, when they are suffering from something (.) that would then be a painkiller for pain or something to relieve constipation for a constipation problem. (P8)

Nurses also view personal skills as essential for holistic, patient-centred, and empathic care. Complementing professional expertise, they are primarily reflected in communication and social competence, such as transparency in interventions and repeatedly explaining care to patients:

Patients need a great deal of explanation and the impression that I am confident in what I am doing (.) so I think communication is very important, explaining what I am doing to the patient at that moment (.) explanation, explanation, and that is also something we train ourselves in (.) many things are well-practised routines for us, very much taken for granted, but for those affected and the people around them, it is completely new territory in a highly sensitive situation (2) please talk, explain, and also offer to explain even more […] so this communicative aspect, I think that is the absolute key element, I am firmly convinced of that. (P12)

Patient-centred communication and needs-based support are highlighted as being important for building trust and facilitating care processes. Reliability, transparent communication, and professional confidence appear to provide patients with guidance and stability, while self-confidence, responsibility, and self-reflection are highlighted as important personal qualities. Projecting confidence, like “I also have to convey that I know what I am doing” (P9) despite inner uncertainty seems to function as a coping strategy to maintain trust, stability, and reassurance for patients and their families in challenging situations:

And it is important that you project confidence when you are with the relatives and the patient (.) so that you are not uncertain yourself, but rather that you, I’ll put it rather casually, somehow project confidence despite complete uncertainty. (P1)

With regard to responsibility and self-reflection, nurses largely emphasized the importance of recognizing and accepting their own professional boundaries in the interest of patient welfare: “After all, I do not do anything unreflective, and I can somehow justify everything that I do (.) if I couldn’t, I wouldn’t do it, and if in doubt, I would get help if I couldn’t see a way out, of course” (P7). Consequently, responsibility and self-reflection appear to support patient safety and high-quality care, with seeking support in complex situations described as an important aspect of reflective practice. Nurses also appear to seek the most accurate assessment possible, particularly when unfamiliar with a patient and their needs. Direct contact seems to facilitate this and enhance their sense of control over the situation:

So, if I simply have the feeling on the phone that I can’t assess the situation, then it simply helps me to say, ´Okay, I’ll come by` (.) and if I’m unsure because I don’t know the patient very well yet, then I’d rather go there one extra time (.) then I come by simply to get a picture of the situation on site, so that I can then act on site. (P1)

Taken together, it is apparently relevant that specialized knowledge is not described in terms of isolated skills, but as the ability to integrate knowledge, experience, communication, and reflective practice to maintain safe and patient-centred care.

### Consequences

Consequences refer to the perceived effects of nurses’ actions, interactions, and strategies in response to the phenomenon, illustrating how these shape their experience of it.

#### Experiencing meaningful work

Experiencing meaningful work is described as arising from taking on expanded responsibilities and independence in nursing practice. The ability to actively help patients and relieve suffering is closely linked to professional self-efficacy, while immediate action in critical situations is seen as helping to reduce pain, provide relief, and prevent feelings of helplessness:

If I imagine now, um, that I were not allowed to make autonomous decisions and act, I wouldn’t be able to help the patient […] the patient, um, would be suffering from the most severe pain, I would somehow have to get hold of a physician who could administer something to this patient (.) so, honestly, I find that thought awful, because I really believe that anyone who has ever had a patient in front of them who was screaming in pain is glad when they have a whitelist and are simply allowed to act (3) so I am glad that we are allowed to do it this way, and I am glad that we are really allowed to act so autonomously. (P16)

Nurses describe fulfillment in providing reassurance, guidance, and a sense of security for families during end-of-life care. Supporting relatives, addressing uncertainties, and fostering trust are experienced as meaningful aspects of their role:

I am glad that we have so many freedoms (.) that we can really say, ´Well, it was so simple to help this family, this person, by going there, with two drops of Neurocil, with two drops of morphine for the shortness of breath` (2) that we are also able to administer the medication and monitor how it works (.) simply to support the relatives, to convey a sense of security, to point out what can happen, what can be expected, where they can get in touch if they are uncertain (.) um, that is, for me, something that feels complete, good, and safe, and simply, so to speak, um, creates a sense of satisfaction. Because I am allowed to make those decisions. Because then it is helpful. (P5)

Beyond providing effective support, nurses perceive independent decision-making and action as enhancing care quality. Perceiving their interventions as contributing to relief, reassurance, and a peaceful situation for those involved fosters fulfillment and emotional reward, while recognition from relatives reinforces their sense of professional effectiveness and the value of their work:

There is always, also, a bit of the question of, how do I do this now? Will I do justice to everyone? So, to the relatives, the patient, to what everyone else expects of me as well (.) of course you are under pressure (.) but if I didn’t enjoy it, then I wouldn’t do it (.) and when I leave the patient’s home and say, ´Now I have worked well, the patient is at peace, the relatives are at peace`, then that is, so to speak, my reward for it (2) and when the relatives say, I mean, I can’t change the fact that the patients die, but when the relatives say, ´Without you we wouldn’t have managed this at home`, then that is, in quotation marks, my reward. (P1)

Experiencing that independent decisions lead to immediate relief, reassurance, and appreciation appears to reinforce nurses’ sense of professional purpose and the value of their contribution. Professional fulfillment thus seems to arise from perceiving the tangible impact of one’s own actions.

#### Navigating ambivalence between empowerment and stressors

Nurses describe this category as a tension between empowerment and burden. This ambivalence appears to arise from balancing competing demands, emotions, and values in patient care. Although independent decision-making and action offer opportunities for professional growth, they may also involve emotional strain, uncertainty, and overwhelm. As a result, taking on expanded responsibilities is experienced as a complex and dynamic interplay of freedom and insecurity:

Both things pop up (.) great joy, I really enjoy it, this freedom, completely (2) so, being allowed to take responsibility, being allowed to make decisions, also being allowed to bear the consequences of them (.) I think it is a great luxury, I really appreciate that in my work (.) and at the same time it involves a great, well, a responsibility, a bit of a burden, that, um, when I am on call I am allowed to make decisions very quickly, but then I also have to. (P5)

Interviewees acknowledge that independent decision-making and action require critical reflection on their own actions in relation to their professional judgment and competence. Looking back, they occasionally describe situations in which consultation with colleagues would have provided greater reassurance, illustrating the tension between acting independently and recognizing when additional support may be beneficial. Beyond the pressure of independent decision-making, nurses identify specific tasks as sources of stress. Even routine procedures, such as medication administration, may become highly burdensome when carried out alone in complex home-care situations:

And yes, I can remember one case, where I injected cortisone because the patient could no longer breathe (.) it was a tumour in the neck area, the patient could no longer swallow, and the tumour was also compressing the trachea, and because of this cortisone that I was allowed to administer, she then really did become visibly better, and the doctor simply said to me, ´Yes, go ahead and do it`, and I was just afraid because I was nervous myself—oh, if this goes wrong now and she dies here, I will think about it for the rest of my life, that I was the one who killed this woman. (P19)

Decisions and actions with potentially irreversible consequences, including death, are described as a major emotional burden. Concerns about being associated with a patient’s death emerged particularly during medication administration in end-of-life care. Even when interventions were clinically indicated and physician-supported, nurses reported uncertainty, emotional vulnerability, and anxiety about their consequences:

There will again be a port access that does not go well, that perhaps introduces some kind of bacteria because it was not properly prepared beforehand (.) um, there will again be the situation where you administer a medication that you are 100% sure is the right one, and the patient reacts paradoxically (2) and, um, there will again be the moment, in the worst case, where you provoke a last-needle effect and then have to live with that (2) I think that is simply part of our field (.) we care exclusively for people who are very seriously ill, close to death, and highly vulnerable. (P12)

The last-needle effect appears to be understood by nurses as a metaphor for the emotional, social, and symbolic significance of administering a final dose of medication. Although the medication itself is not considered the cause of death, the close temporal relationship between injection and death may create the impression that it was:

One explanation [for the last-needle effect] is that the patient who is very close to death does not die because of the administration of morphine, but rather that the pain releases so much adrenaline that it pushes the body on (2) and when I then treat that pain adequately, the adrenaline subsides, and then you actually see how far the person already is on the way […] but the picture that I present to the relatives is that the nurse comes in, injects something, and the patient dies, and sometimes you still have the syringe, the needle, in your hand, so that is where the term comes from; that is, um, um, an unimaginably awful feeling. (P12)

When nurses must act independently while confronting severe suffering and the limits of symptom control, feelings of overwhelm may arise. They describe tensions between the responsibility to relieve suffering and uncertainty about the effectiveness, ethical justification, and personal burden of further interventions. Consequently, independent decision-making and action are experienced as both a necessity and an emotional burden, requiring a constant balance between responsibility, uncertainty, and helplessness:

Yes, there was a patient with pain, she was lying there and shouting, ´Help, help`, and then, after consulting with the physicians, I administered everything possible to her […] she didn’t feel any better (.) and those are situations where we have to wait […] sometimes that is a very long time, but when you also have the feeling that it isn’t working and I then have to give something again, where in the end you reach the point of saying, ´I don’t know what else I can give her […] and I don’t know what else I can take responsibility for if this doesn’t get any better’, those are situations where the patients are really desperately asking for help because they are experiencing massive pain episodes that you simply cannot get under control. (P1)

Such situations are described as exposing the limits of professional action despite appropriate clinical management. Rather than reflecting a lack of competence, these experiences appear to illustrate the emotional challenge of remaining present and sharing patients’ and relatives’ distress when suffering cannot be alleviated immediately. While increased responsibility fosters professional self-confidence and meaningful work, it may also generate pressure, uncertainty, and concerns about doing the right thing. Thus, it seems that navigating ambivalence is experienced as a continual balancing of self-determination and vulnerability amid conflicting demands, emotions, and expectations.

### Context

Contextual factors of the core phenomenon describe the structural and situational conditions nurses encounter in SOPC. These arise from managing complex and unstable symptoms in patients’ homes and from the close, ongoing contact with patients and their families, which fosters a strong sense of commitment.

#### Managing complex care situations in patients’ homes independently

For nurses, working in SOPC involves managing complex care situations in patients’ homes, often without immediate support from colleagues or physicians, particularly when responding to rapidly changing patient needs. These situations require prompt symptom-oriented interventions as well as the ability to address challenging behaviours, potential risks, and the emotional strain on family members. The home setting and the presence of family members further appear to create additional emotional and professional demands:

I think that, in SOPC, this major factor is added, namely that it takes place outside the hospital (.) that, when there is uncertainty or a complication, you cannot simply call a colleague over in the quickest way (1) and even with routine tasks, um, that you carry out confidently […] it can happen that even with a correctly performed port access you might still trigger a complication […] so even with the confidence that you have done nothing wrong professionally, um, um, it happens in the home setting and usually in very emotionally intense situations (1) perhaps there are relatives standing nearby with very high expectations (.) and then to have the standing and the confidence to manage that crisis, that is another completely different factor. (P12)

So, professional confidence can be understood as a prerequisite for taking on responsibilities in complex care situations. Furthermore, independent decision-making and action are described as extending beyond symptom-oriented care to encompass psychosocial, everyday-life, and relational aspects. Nurses also appear to develop therapeutic approaches and contribute actively to interprofessional decision-making beyond traditional role boundaries:

When I have the care of a patient whose situation is very complex, so especially in outpatient palliative care, with regard to, um, the experience of pain and suffering, um, then this multidimensionality is often the background against which we think […] and there I certainly do see different nursing perspectives (2) from non-pharmacological interventions, the provision of assistive devices, and a person’s management of everyday life in their home environment (.) how do they move around? How are they socially connected? (P3)

At the same time, an independent professional role is emerging, characterized by independent decision-making, the need to understand patients’​​ situations holistically, and active involvement in interprofessional care planning.

#### Being strongly committed to patients and their families

Strong commitment to patients and their families appears closely linked to the core phenomenon, as nurses’ relationship-oriented approach may foster their willingness to take on expanded areas of responsibility despite the risks and uncertainties of independent decision-making and action. Interviewees describe how frequent contact in patients’ homes promotes close, trusting relationships with patients and family members, enabling nurses to recognize and respond to needs that may not be explicitly expressed. Empathy is considered central to this approach, facilitating trust and the recognition of unspoken concerns through attentiveness and emotional openness:

I have a good capacity for empathy, and I also rely on that (.) so I think I have a high level of empathy for what is there on site (.) so sometimes it really is the case that, um, I open all my antennas, that I am completely open with the patient, so that I can really hear and feel the nuances as well (2) who is standing together with whom, where? What is really important now, and what is it? (P1)

Interviewees further describe that the ability to perceive unspoken concerns and underlying needs and to attend to subtle verbal and non-verbal cues appears to support a deeper understanding of patients’ situations while also highlighting the emotional demands associated with maintaining this level of sensitivity in everyday practice. Being strongly committed to patients and their families appears to involve caring deeply and building trusting relationships with patients, combining professional competence with empathy and emotional engagement:

I had a very close relationship with a patient who is now in a hospice […] she had her own attitudes, shall I say, and I like people like that because I’m like that too (laughs) (.) this patient always maintained her composure, and at first I had thought that we wouldn’t really warm to one another […] and you could really notice how, little by little, trust developed (.) then there was, um, the situation where the pain became worse […] and then I said to my colleagues, ´I would like to go there again because I think her pain medication is not adequately covering her` (.) um, my colleague then said that he had already been there and that she did not want an increase in the fentanyl patches (.) I went there anyway, and it really was the case that she let me apply more patches. (P13)

Overall, the findings suggest that for nurses working in SOPC, it is not only about professional competence but also about building interpersonal relationships to support patients. From a nursing perspective, this appears to be an essential requirement and an important component of patient care.

## Discussion

This study examines how nurses working in SOPC experience taking on expanded responsibilities and performing independent decision-making and action. The findings illustrate that independent decision-making and action are closely linked to trusting interprofessional relationships, supportive team structures, and strong engagement in patient care. Consequently, rather than representing professional independence from others, autonomy emerges as a relational achievement that is enabled through trust, recognition, and ongoing interprofessional interaction.

The contribution of this study lies in showing how independent nursing practice, responsibility, and relational embeddedness intersect in SOPC. Rather than experiencing autonomy as acting independently of others, nurses described being able to make independent decisions and take action because they were embedded in trusting interprofessional relationships and could access support when needed. Particularly in situations where physicians were not directly present, autonomy involved the ability to act independently and the willingness to assume responsibility for these decisions and to recognize when additional expertise was required. Thus, relational support and consultation did not appear to diminish autonomy but enabled nurses to exercise it safely.

Traditional understandings of professional autonomy emphasize independence and self-governed decision-making [13, 14]. In contrast, our findings align with concepts of relational autonomy [38], which view autonomous action as embedded within social relationships and supportive professional networks. From this perspective, autonomy does not arise despite professional interdependence but is enabled through it. In this context, trust emerges as a key factor that develops through a gradual relational process.

Existing descriptions of trust within interprofessional teams emphasize that trust grows from ongoing collaboration and mutual understanding of competences laying the foundation for psychological safety [39–41]. This is reflected in our findings, as interviewees highlight trust as a growing process through repeated interactions, shared experiences and demonstrated competences. This process resembles the concept of Entrustable Professional Activities (EPAs) [42], which conceptualizes professional autonomy as emerging from the willingness of others to entrust individuals with responsibilities based on demonstrated competence, reliability, and professional judgement. From this perspective, the trust described by participants can be understood not merely as an interpersonal aspect but also as a mechanism through which greater clinical responsibility is progressively granted. However, entrustment in SOPC seems to extend beyond competence-based delegation. Trust is described as being co-constructed through ongoing relationships, emotional support, accessibility of colleagues, and opportunities for shared reflection. This may create conditions for coordinated decision-making, effective teamwork, and the provision of safe and high-quality care [41]. From a psychological and practical perspective, trust within interprofessional teams functions as a „safety net“ [43 p.5], particularly in complex and high-risk care situations. Trust within SOPC teams may operate through mechanisms of psychological safety, enabling nurses to express uncertainty, seek support, and disclose vulnerability without fear of professional judgement. Consequently, supportive structures enabling nurses to perform their roles safely and effectively are essential for autonomy in nursing practice.

Concepts of collective leadership [44] locate responsibility and decision-making within shared expertise rather than formal hierarchical authority. This interpretation aligns with literature on interprofessional collaboration, which emphasizes participative decision-making, shared knowledge and expertise, mutual trust, and the reduction of hierarchical barriers [45–47]. These aspects are reflected in the core phenomenon, as nurses experience autonomy in practice not as an isolated activity but as part of an ongoing dialogue among professional equals.

Beyond this relational dimension, the findings highlight responsibility under uncertainty as a central aspect of nurses’ experiences of autonomy in SOPC. Accordingly, nurses experience responsibility in relation to the consequences of their decisions and actions and their immediate impact on patients. This is particularly apparent in situations in which the need to relieve suffering is accompanied by uncertainty about possible outcomes. The “last-needle effect” illustrates this tension, as even clinically justified actions may carry considerable emotional weight when they are closely associated with deterioration or death. In this sense, bearing responsibility emerges as an integral, but potentially burdensome, dimension of experiencing expanded responsibilitites by nurses in SOPC.

In addition, the findings highlight tensions between expanded nursing responsibilities and their formal authorization. Interviewees described situations in which they acted on their own clinical judgment due to emergencies, including decisions extending beyond predefined treatment, while also reporting uncertainty regarding the boundaries of delegated practice. Importantly, these accounts do not allow conclusions about the legal permissibility of individual actions, but they indicate that formal authorization and accountability were not always perceived or implemented consistently in SOPC practice. This ambiguity is particularly relevant in situations involving medication administration and other interventions with potentially serious consequences. Thus, strengthening nurses’ confidence in their own practice requires professional competence and interprofessional trust, but also clearly defined scopes of practice, delegation processes, and accountability structures to support nurses and create conditions perceived as important for safe patient care. While trust and collaborative decision-making can strengthen independence in nursing practice, they are also important in lessening challenges associated with managing complex care situations. Alongside empowerment, experienced autonomy in nursing practice within SOPC appears as a balancing act between independence and burden. Overall, these findings suggest that expanded nursing responsibilities require organizational structures that provide accessible professional support, opportunities for consultation and shared reflection, as well as clear pathways in situations of uncertainty. Such structures may contribute to nurses’ emotional safety and to safe clinical decision-making. Prior studies [48–50] show that palliative care nurses experience stress, e.g., when feeling inadequately prepared. To prevent and address distress, supervision should additionally be structurally integrated into team structures and training programs to strengthen existing resources and individual resilience [51]. However, given the diversity of supervision approaches, the chosen format should be specifically aligned with the needs of palliative care nurses and the intended outcomes, such as emotional support and reflective practice.

Healthcare organizations should actively promote trusting interprofessional relationships, clearly defined roles as well as responsibilities, and supportive team structures that enable shared decision-making in nursing practice. The implementation of expanded nursing roles should therefore be understood as a structural and educational process as well as a relational one, in which trust, mutual recognition, and continuous collaboration are essential prerequisites.

Particularly in palliative care, nurse-led care models have gained increasing attention as a response to growing demand, ageing populations, rising chronic illness, and shortages of specialist physicians [52–55]. These models involve expanded nursing roles encompassing assessment, symptom management, psychosocial support, patient and family education, care coordination, and advance care planning [52]. Evidence suggests that nurse-led palliative care can improve symptom control in care situations, emotional well-being, communication, quality of life, continuity of care, access to services, family involvement, and, particularly in community and home-care settings, reduce healthcare utilisation [54]. Although the present findings were derived specifically from nurses working in German SOPC, they may offer insights into relational and organizational conditions that are also discussed in the context of nurse-led care models [17]. These models represent a broader shift toward greater use of nursing expertise and recognition of nurses as autonomous providers within interdisciplinary and multiprofessional (palliative) care teams [53, 54]. This is particularly relevant in healthcare systems where advanced nursing roles are still evolving and being implemented selectively [56, 57].

In addition, some of the competences, responsibilities, and functions described by nurses working in SOPC overlap with characteristics associated with advanced nursing roles [58], including independent clinical judgment, responsibility for complex care situations, and the application of specialized knowledge. These findings suggest that characteristics discussed in the context of advanced nursing practice may also be reflected in the everyday practice of experienced nurses in SOPC. Against this background, nurse-led and advanced nursing models provide relevant contexts in which our findings may be considered. However, similarities in competences and responsibilities should be distinguished from formal role equivalence, as advanced nursing practice requires specific educational requirements that were not defining characteristics of the nurses included in this study. The experiences described in this study show that nurses with extensive work experience and specialist palliative care training can manage complex care situations independently. In this context, nurse-led models may provide a framework for enabling qualified nurses to assume greater clinical responsibility in palliative care. However, our findings suggest that such models require clearly defined scopes of practice, formal authorization and accountability structures, as well as accessible interprofessional support to ensure that expanded responsibilities can be assumed safely and sustainably. This is particularly relevant in Germany, where interviewees perceived legal gray areas and uncertainties regarding the delegation and formal authorization of specific nursing activities. Establishing clearer regulatory frameworks may therefore be important for the further development and implementation of expanded and nurse-led roles in Germany.

### Limitations

The study included nurses working in SOPC in Germany. As SOPC represents a highly specialized care setting characterized by expanded nursing responsibilities and close interprofessional collaboration, the legal, organizational, and professional context of the German healthcare system, where advanced nursing roles and nurse-led models are still emerging, needs to be considered. However, although the organizational and legal framework is specific to Germany, the explanatory model developed in this study focuses on relational and organizational processes that are likely to extend beyond this context. Trust, professional competence, interprofessional collaboration, and responsibility have been described as central components of autonomous nursing practice across different healthcare systems. Therefore, the findings may inform the implementation and development of advanced nursing roles and nurse-led care models internationally, while acknowledging that their transferability should be considered in relation to local organizational and regulatory conditions.

A further limitation concerns the sampling strategy based on practical considerations, such as accessibility, availability, and willingness to participate [35]. Accordingly, theoretical sampling in the classical sense of grounded theory methodology was not conducted, and the iterative collection of additional data depended on how interviews could be scheduled. It can therefore be assumed that the sampling strategy limited the possibility of deliberately recruiting additional participants to further elaborate, differentiate, or challenge emerging categories.

The identified relational and organizational conditions may reflect contextual factors that differ from countries with more established advanced nursing practice roles.

The sample was characterized by a high level of professional experience and work experience in SOPC and all participants were certified palliative care nurses. This should be considered when interpreting the findings, as the experiences of less experienced or differently qualified nurses, particularly those newly assuming expanded responsibilities, may differ in terms of autonomy, responsibility, and trust across levels of expertise and career stages.

While the use of a semi-structured interview guide ensured consistency across interviews and coverage of key topics, it may have shaped participants’ narratives and issues they emphasized during data collection. As a result, aspects of the phenomenon under study not addressed in the guide may have received less attention. In addition, as responsibility and interprofessional collaboration were included as predefined topic areas in the interview guide, the prominence and relationships of these concepts in participants’ quotations may partly reflect the structure of data elicitation.

Although the data were considered sufficiently rich to address the research question, only the perspectives of nurses were included. Views of physicians, patients, family members, and other healthcare professionals on autonomy in nursing practice and respective relational conditions supporting it were not explored.

Lastly, the term ​autonomy​ was used in the interview guide without providing participants with an explicit definition, allowing for individual associations and interpretations of the concept. This openness should be considered in relation to the more specific use of the concept in our findings, as participants’ responses were based on their own understanding of autonomy rather than on a predefined conceptualization.

## Conclusions

Taking on expanded responsibilities, including independent decision-making and action, is primarily experienced by nurses working in SOPC as a collaborative form of autonomy embedded in trusting professional relationships and supportive team structures. The ability to make independent decisions and take on expanded responsibilities depends on personal and professional competence, but equally on interprofessional recognition, safety, and opportunities for collaborative reflection.

In addition, the findings broaden existing concepts of relational autonomy by showing that nurses in SOPC experience taking on expanded responsibilities and acting under uncertainty more positively when they feel supported by trusting interprofessional relationships. Consequently, organizational structures, leadership, institutional support, and practical frameworks contribute to the development and maintenance of trust as a key factor of autonomous nursing.

The perceived consequences of trust are similarly multifaceted, affecting individual well-being, professional role development, team functioning, collaboration, and decision-making, as well as nurses’ perceptions of patient care. Trust should therefore be understood as a relational and context-dependent phenomenon that is continuously negotiated within healthcare environments.

While existing research on advanced nursing practice and nurse-led care predominantly focuses on patient outcomes and competence profiles, less is known about how nurses experience autonomy in different healthcare settings. The present findings contribute to this discussion by illustrating how experienced nurses in SOPC perceive and live autonomy, responsibility, and the relational conditions that support independent clinical judgment. Future research could examine whether and how these conditions are reflected in formally defined advanced nursing and nurse-led roles.

## Supplementary Information

Supplementary Material 1: Additional File 1. Semi-structured interview guide. Description: Topic areas, associated guiding questions and stimulants to promote the narrative process during interviews.

## Data Availability

The original dataset generated and analyzed during the current study is not publicly available because participants’ informed consent did not include the sharing of original data. Therefore, the original data cannot be made available upon request.

## Abbreviations

SOPC: Specialized Outpatient Palliative Care

## Footnotes

1 Whisper Transkription [Version 12.18.2 (1292)]

2 GESIS-Leibniz Institute for the Social Sciences.
