# Supplementary material for: Nurses’ experiences of expanded responsibilities, independent decision-making and action in specialized outpatient palliative care: a qualitative interview study
Source: BMC Palliat Care. 2026 Oct 30;25:330. doi: 10.1186/s12904-026-02399-0 (PMC13729503; doi:10.1186/s12904-026-02399-0)
Supplement: Supplementary file 1 [file 12904_2026_2399_MOESM1_ESM.pdf]

Additional File 1: Semi-structured interview guide

Topic area	Guiding questions & stimulants
1. **Experiences with interprofessional collaboration in daily practice.**	Please think of situations in your daily practice that you would describe as examples of good care. What makes these situations particularly positive, and why?<br><br>Are there any processes or procedures in healthcare that you would like to see changed or improved? If so, which ones and why?<br><br>Please tell me about your experiences of working with physicians?<br><br>**<u>Stimulants</u>**<br>• Please describe a specific situation in which teamwork worked particularly well? What made it work well?<br>• Please describe a specific situation in which teamwork was particularly challenging? What made it challenging?
2. **Experience with taking on expanded responsibilities in daily practice.**	When you hear the phrase "taking on expanded responsibilities in day-to-day practice," what comes to mind?<br><br>Please think of situations in your daily practice in which you take on expanded responsibilities. How do you experience these situations? Please feel free to describe specific examples that are particularly important to you in as much detail as you wish.<br><br>**<u>Stimulants</u>**<br>• What factors lead you to take on expanded responsibilities?<br>• What makes it easier for you to take on expanded responsibilities?<br>• What do you need in order to take on expanded responsibilities successfully?<br>• What makes it more difficult for you to take on expanded responsibilities?
3. **Experiences with independent decision-making and action in daily practice.**	Please think of specific situations in patient care in which you make independent decisions and act autonomously. Please describe these situations in detail.<br><br>What helps you make independent decisions and act autonomously?

	What makes it difficult for you to make independent decisions and act autonomously?<br><br>Are there situations in which you are unable to make independent decisions and act autonomously? If so, why?<br><br>What would help you make decisions and act independently?<br><br>Which personal skills or qualities help you make decisions and act independently?
4. **Experiences with taking on tasks delegated by physicians in daily practice.**	Please think of situations in which you carry out tasks delegated by physicians. Please describe these situations and tasks in detail.<br><br>How do you experience these situations in terms of responsibility?<br><br>Which delegated tasks do you find more comfortable or less comfortable to perform, and why?<br><br>What helps you when carrying out tasks delegated by physicians?<br><br>What makes it difficult for you to carry out tasks delegated by physicians?<br><br>Please think of a specific situation in which you felt overwhelmed while carrying out a task delegated by a physician. Please describe what happened.<br><br>**Stimulants**<br>• What would have helped you in this situation?<br>• What sources of support or resources did you draw on in this situation?<br>• Looking back, is there anything you would do differently? If so, why?
5. **Recommendations for nurse-led care.**	In your opinion, what knowledge, skills, and personal qualities do nurses need to care independently for patients with complex care and treatment needs? What is needed for nurses to work autonomously with one another, for example, when initiating a consultation with another healthcare professional?
